# Association between lipoprotein(a) and coronary heart disease risk in type 2 diabetes mellitus and evaluation of statin treatment effects

**DOI:** 10.1590/1806-9282.20240870

**Published:** 2025-03-17

**Authors:** Li Li, Liwu Xu

**Affiliations:** 1 Bengbu Medical University, School of Graduate, Bengbu, People's Republic of China.; 2 Anhui University of Science and Technology, The First Affiliated Hospital, Department of Endocrinology – Huainan, People's Republic of China.

**Keywords:** Diabetes, Lipoprotein(a), Coronary heart disease, Residual cardiovascular risk, Rosuvastatin

## Abstract

**OBJECTIVE:**

The aim of this study was to investigate the association between lipoprotein(a) and coronary heart disease risk in type 2 diabetes mellitus patients and evaluate the effectiveness of statin therapy.

**METHODS:**

This retrospective analysis included 120 patients diagnosed with type 2 diabetes mellitus. Of these, 90 patients diagnosed with coronary heart disease via coronary angiography received rosuvastatin treatment for over 6 months. The remaining 30 patients exhibited no coronary heart disease or other diabetic macrovascular complications and had not received any lipid-lowering treatment. Patients with type 2 diabetes mellitus and coronary heart disease were categorized into two groups based on the severity of coronary lesions. Baseline characteristics and blood lipid data were compared among groups. Logistic regression analysis was employed to investigate the association between lipoprotein(a) and coronary heart disease risk in type 2 diabetes mellitus patients. Receiver operating characteristic curves were utilized to evaluate the diagnostic value of lipoprotein(a) for coronary heart disease.

**RESULTS:**

Compared with the control group, lipoprotein(a) levers were higher in both the mild and severe groups. Logistic regression analysis demonstrated that lipoprotein(a) is independently associated with the risk of coronary heart disease in type 2 diabetes mellitus patients. The area under the receiver operating characteristic curve for lipoprotein(a) was 0.729. When lipoprotein(a) was 97.5 mg/L, the diagnosis of coronary heart disease had high sensitivity and specificity. After statin therapy, high-density lipoprotein cholesterol and apolipoprotein A levels increased, while other lipid parameters decreased. However, the lipoprotein(a) level decrease was not statistically significant.

**CONCLUSION:**

Lipoprotein(a) is an independent risk factor for coronary heart disease in type 2 diabetes mellitus patients. Lipid-lowering therapy with statins alone cannot reduce lipoprotein(a) levels.

## INTRODUCTION

Type 2 diabetes mellitus (T2DM), as the risk factor for cardiovascular disease (CVD), often coexists with coronary heart disease (CHD), cerebrovascular disease, and lipid metabolism disorders^1^. Lipoprotein(a) [Lp(a)], a unique lipoprotein synthesized in the liver, comprises low-density lipoprotein-like particles, apolipoprotein A (apoA), and apolipoprotein B (apoB) connected by covalent bonds^2^. Lp(a) contributes to thrombus formation by inhibiting fibrinolysis and serves as an independent risk factor for atherosclerotic CVD^3^. Studies indicate that Lp(a) is associated with early-onset atherosclerosis in T2DM patients^4^. However, further investigation is necessary to fully understand the link between Lp(a) levels and CHD risk in T2DM patients.

Statins, commonly prescribed to lower low-density lipoprotein cholesterol (LDL-C) levels and prevent cardiovascular events in CHD patients, have limitations. Studies highlight the persistent residual risk for CHD despite statin therapy. Lp(a) has been identified as an important residual risk factor^5^. This study aims to investigate the association between Lp(a) and CHD risk in T2DM patients. It also seeks to observe the effect of statin treatment on Lp(a) levels. The findings will offer insights into optimizing lipid-lowering treatment strategies and reducing CHD risk in T2DM patients.

## METHODS

### Study population

This retrospective analysis comprised 90 patients (56 males and 34 females) diagnosed with T2DM and CHD at the First Affiliated Hospital of Anhui University of Science and Technology between January 2018 and December 2023. These patients, constituting the case group, had received a nightly oral dose of 10 mg of rosuvastatin calcium tablets (IPR Pharmaceuticals, Incorporated, Canovanas, Puerto Rico) for over 6 months. None had used any lipid-lowering therapy before starting statin treatment. Based on coronary angiography results, patients in the case group were divided into distinct groups: a mild group with single- or double-vessel disease (36 cases) and a severe group with multivessel disease (54 cases). Additionally, during the same period, 30 T2DM patients without CHD or other major diabetic vascular complications, including 18 males and 12 females, who had not received any lipid-lowering therapy were included in the control group.

Inclusion criteria were defined as follows: (1) compliance with the diagnostic criteria for T2DM outlined in the 2016 ADA guidelines; (2) diagnosis of CHD based on coronary angiography results from our hospital, with luminal stenosis exceeding 50% as the standard; (3) receipt of statin therapy for a minimum of 6 months following the diagnosis of CHD; and (4) patients with complete clinical data. Exclusion criteria included(1) patients with incomplete data; (2) liver or kidney insufficiency; (3) concurrent hypothyroidism or other conditions affecting blood lipid levels; and (4) the presence of malignant tumors, immune system diseases, or severe mental illnesses.

### Data collection

Baseline characteristics of the patients, including gender, age, and lipid parameters, were retrieved by reviewing the electronic medical record system. Moreover, data on total cholesterol (TC), TG, HDL-C, non-high-density lipoprotein cholesterol (non-HDL-C), LDL-C, Lp(a), apoA, and apoB were collected both before and after 6 months of statin therapy in the case group.

### Statistical analysis

Data analysis was performed using IBM SPSS Statistics version 25.0. Quantitative data following a normal distribution were presented as mean±standard deviation (SD), and group comparisons were performed using t-tests or analysis of variance. Quantitative data not following a normal distribution were expressed as median with interquartile ranges, and group comparisons were performed using nonparametric tests. Numbers (n) and percentages (%) were used to describe categorical variables, and chi-square tests were used for group comparisons. Univariate and multivariate logistic regression analyses were performed to explore the association between Lp(a) and CHD risk in T2DM patients. Receiver operating characteristic (ROC) curve analysis was employed to evaluate the diagnostic value of Lp(a) for CHD. A p<0.05 was considered statistically significant.

## RESULTS

### Comparison of baseline characteristics among the mild, severe, and control group

Table 1 reveals significant differences in age, a history of hypertension, and Lp(a) among the three groups (p<0.05). Compared with the control group, patients in the severe group were older (p<0.05). Additionally, Lp(a) levels and the prevalence of hypertension were notably higher in both the mild and severe groups compared with the control group (p<0.05). Nevertheless, no significant differences were observed in gender, diabetes duration, TG, TC, HDL-C, LDL-C, non-HDL-C, apoA, and apoB among the three groups.

**Table 1:** Baseline characteristics of study participants.

Characteristic		Control (n=30)	Mild (n=36)	Severe (n=54)	p-value
Age (years)		56.43±8.69	61.64±10.01	64.41±10.40*	0.003
Gender (male/female)		18/12	25/11	31/23	0.504
Hypertension (%)					
	No	21 (70.00%)	12 (33.33%)	14 (25.93%)	<0.001
	Yes	9 (30.00%)	24 (66.67%)*	40 (74.07%)*	
Diabetes duration (years)		5.00 (1.00,8.50)	4.00 (1.00,6.75)	5.50 (2.00,10.00)	0.471
Lp(a) (mg/L)		59.00 (38.00,83.75)	136.50 (42.50,309.25)*	154.00 (64.75,325.50)*	0.001
TG (mmol/L)		1.45 (0.82,3.60)	1.74 (1.36,2.87)	1.66 (1.20,2.40)	0.442
TC (mmol/L)		4.12±1.28	4.04±0.91	4.27±1.09	0.599
HDL-C (mmol/L)		0.97±0.26	0.85±0.21	0.87±0.25	0.089
LDL-C (mmol/L)		2.42±0.85	2.67±0.92	2.70±0.85	0.368
Non-HDL-C (mmol/L)		3.15±1.28	3.19±0.87	3.41±1.05	0.489
ApoA (g/L)		1.19±0.21	1.13±0.18	1.11±0.21	0.242
ApoB (g/L)		0.73±0.17	0.78±0.20	0.81±0.21	0.181

* Compared to the control group, p<0.05.

Lp(a): lipoprotein(a); TG: triglycerides; TC: total cholesterol; HDL-C: high-density lipoprotein cholesterol; LDL-C: low-density lipoprotein cholesterol; non-HDL-C: non-high-density lipoprotein cholesterol; ApoA: apolipoprotein A; ApoB: apolipoprotein B.

### Risk factors for coronary heart disease in type 2 diabetes mellitus patients

The results of the univariate logistic regression analysis indicated a significant association among age (OR 1.071, 95%CI 1.024–1.119; p=0.002), a history of hypertension (OR 5.744, 95%CI 2.325–14.186; p< 0.001), Lp(a) (OR 1.011, 95%CI 1.004–1.019; p=0.002), and an increased risk of CHD in T2DM patients. Conversely, HDL-C (OR 0.170, 95%CI 0.032–0.892; p=0.036) exhibited a protective effect against CHD. A multivariate logistic regression analysis revealed that Lp(a) was independently associated with the risk of CHD in T2DM patients after adjusting for other indicators (Table 2).

**Table 2:** Risk factors for coronary heart disease in type 2 diabetes mellitus patients by logistic regression analyses.

		Univariate model			Multivariate model		
		OR	95%CI	p	OR	95%CI	p-value
Age (years)		1.071	1.024–1.119	0.002	1.038	0.983–1.096	0.175
Gender (male/female)		1.098	0.471–2.558	0.828			
Hypertension (%)							
	No	1.000	Reference		1.000	Reference	
	Yes	5.744	2.325–14.186	<0.001	5.672	1.897–16.958	0.002
Diabetes duration (years)		1.012	0.949–1.078	0.720			
Lp(a) (mg/L)		1.011	1.004–1.019	0.002	1.012	1.003–1.020	0.006
TG (mmol/L)		0.969	0.792–1.185	0.759			
TC (mmol/L)		1.051	0.713–1.550	0.800			
HDL-C (mmol/L)		0.170	0.032–0.892	0.036	0.074	0.009–0.606	0.015
LDL-C (mmol/L)		1.432	0.868–2.364	0.160			
ApoA (g/L)		0.181	0.023–1.413	0.103			
ApoB (g/L)		6.972	0.779–62.359	0.082			

Lp(a): lipoprotein(a); TG: triglycerides; TC: total cholesterol; HDL-C: high-density lipoprotein cholesterol; LDL-C: low-density lipoprotein cholesterol; ApoA: apolipoprotein A; ApoB: apolipoprotein B; OR: odds ratio; CI: confidence interval.

### Receiver operating characteristic curve analysis of the diagnostic value of lipoprotein(a) for coronary heart disease

We conducted the ROC curves analysis to evaluate the diagnostic value of Lp(a) for CHD. Lp(a) (area under the receiver operating characteristic curve [AUROC]: 0.729, 95%CI 0.641–0.817, cutoff value: 97.5 mg/L, sensitivity: 64.4%, specificity: 86.7%; p<0.001) had a specific diagnostic value for CHD.

### Comparison of lipid parameters before and after six months of statin therapy in the case group

To assess the effect of statin therapy, we compared the lipid parameter levels before and after 6 months of treatment in the case group. Following treatment, there was a significant decrease in TG, TC, LDL-C, non-HDL-C, and apoB levels (p<0.001), accompanied by an increase in HDL-C and apoA levels (p<0.05). Lp(a) levels decreased, but the difference had no statistical significance (Table 3).

**Table 3:** Changes in lipid parameters following statin therapy.

Lipid parameters	Baseline	6 months	p-value
TG (mmol/L)	1.70 (1.31,2.57)	1.57 (1.12,2.14)	<0.001
TC (mmol/L)	4.18±1.02	3.45±1.13	<0.001
HDL-C (mmol/L)	0.86±0.24	0.91±0.27	0.006
LDL-C (mmol/L)	2.68±0.87	2.01±0.94	<0.001
Non-HDL-C (mmol/L)	3.32±0.99	2.53±1.07	<0.001
Lp(a) (mg/L)	152.00 (51.75,319.75)	104.00 (51.25,314.25)	0.224
ApoA (g/L)	1.12±0.20	1.16±0.22	0.049
ApoB (g/L)	0.80±0.21	0.66±0.25	<0.001

TG: triglycerides; TC: total cholesterol; HDL-C: high-density lipoprotein cholesterol; LDL-C: low-density lipoprotein cholesterol; Non-HDL-C: non-high-density lipoprotein cholesterol; Lp(a): lipoprotein(a); ApoA: apolipoprotein A; ApoB: apolipoprotein B.

## DISCUSSION

In recent years, there has been a significant rise in the incidence of T2DM, accompanied by an increased risk of mortality due to cardiovascular complications associated with diabetes^6^. This trend has prompted researchers to explore novel and established biological markers for assessing cardiovascular risk to facilitate early intervention^7^. Particularly, there is a growing interest in the potential role of Lp(a) in promoting cardiovascular events. Numerous studies have demonstrated that elevated Lp(a) is an independent risk factor for CHD^8^. A prospective cohort study substantiated that Lp(a) levels are independently linked to the risk of adverse cardiovascular outcomes among T2DM patients experiencing cardiovascular events^9^. Chen et al.^4^ conducted research on the role of Lp(a) in the development of early-onset atherosclerosis in T2DM patients and concluded that increased Lp(a) levels pose a risk factor for carotid atherosclerosis in this demographic. This research revealed a significant elevation in Lp(a) levels among T2DM patients with CHD compared with those without CHD. The findings suggest that increased Lp(a) levels contribute to the risk of developing CHD in T2DM patients. Multivariate logistic regression analysis further confirmed that Lp(a) was independently associated with the risk of CHD in T2DM patients, likely due to the role of Lp(a) in atherosclerosis and thrombus formation. The mechanism may involve (1) direct deposition of Lp(a) on endothelial cells, promoting lipid uptake by macrophages and accelerating atherosclerosis; (2) induction of endothelial dysfunction by high levels of Lp(a); and (3) inhibition of fibrinolysis through competition with plasminogen while inhibiting tissue factor pathway inhibitor to promote thrombosis^10^. Although no significant differences in LDL-C, TG, TC, and HDL-C levels were observed between the CHD group and the diabetic group without CHD, the limited sample size in the analysis may have contributed to these findings. Additionally, lipolysis is increased in patients from the diabetic group without CHD due to insulin resistance. This process results in elevated triglyceride synthesis and fatty acid release, leading to higher levels of LDL-C and lower levels of HDL-C^11^.

The ROC curves analysis expressed that Lp(a) had a superior diagnostic value for CHD, with an AUROC exceeding 0.70. An Lp(a) level exceeding 97.50 mg/L indicates an increased CHD risk in T2DM patients. Notably, these threshold values were lower than those recommended by national guidelines for predicting CHD risk, which typically suggest an Lp(a) level exceeding 30 mg/dL (or 300 mg/L). The consensus from the European Atherosclerosis Society emphasizes that elevated Lp(a) levels pose a persistent threat to cardiovascular events, even when LDL-C is controlled at a relatively low level^12^. Additionally, cardiovascular risk increases with higher Lp(a) levels^12^. Given the consistent relationship between Lp(a) concentration and cardiovascular risk, the consensus statement suggests incorporating Lp(a) levels of ≥30–50 mg/dL into cardiovascular risk management^12^. However, our findings indicated an even lower critical value for Lp(a)-related CHD risk in T2DM patients, further affirming high Lp(a) levels as a cardiovascular risk factor in this group.

According to the 2023 Guidelines for Lipid Management in China, LDL-C is identified as the primary target for lipid intervention, with non-HDL-C as the secondary target^13^. Additional intervention indicators include TG, Lp(a), and apoB^13^. Similarly, the European Society of Cardiology and the European Society of Atherosclerosis emphasize the importance of adjusting LDL-C, non-HDL-C, and Lp(a) levels to reduce cardiovascular risk^14^. The results of our retrospective study demonstrated significant improvements in lipid parameters among T2DM patients with CHD following statin therapy, except for Lp(a). Currently, there is no international consensus on the impact of statins on Lp(a) levels^15^. Some meta-analyses have suggested the benefits of statin therapy in ameliorating Lp(a) levels, yet others indicate statins may increase Lp(a) levels^10^. Our study found that while Lp(a) levels decreased after statin treatment, the change was not statistically significant. The potential reasons are as follows: (1) approximately 70% to ≥90% of Lp(a) levels are genetically determined; the genetic variation in apoA subtypes can lead to significant changes in Lp(a) levels^16^; (2) statins can lower cholesterol levels in hepatocytes by inhibiting cholesterol synthesis and stimulate the upregulation of LDL receptors on the hepatocyte surface; this process accelerates the catabolism of apoB-containing lipoproteins, thereby reducing Lp(a) levels^17^. However, statins can also increase the synthesis and secretion of Lp(a) mRNA and apoA, leading to an elevation in Lp(a) levels^18^. Previous research has shown that niacin can significantly decrease Lp(a) levels^19^. However, the latest consensus advises against niacin due to the lack of clear clinical benefits^12^. Proprotein convertase subtilisin/kexin type 9 inhibitors are known to lower Lp(a) levels by inhibiting hepatic absorption, and lipoprotein fractionation can be used for patients with high Lp(a) levels and progressive CVD^20^. There is yet to be a consensus on the optimal target value of Lp(a). Therefore, in line with most guidelines, early screening for high Lp(a) levels and implementing intensive risk factor management such as blood glucose, blood pressure, and LDL-C levels are recommended for clinical benefits.

Due to the inclusion of retrospective case data from the cardiovascular ward in this study, certain indicators, such as pancreatic function and blood glucose control in T2DM patients, are missing. Additionally, the limited sample size necessitates further prospective, multicenter studies to reach more definitive conclusions. Despite these limitations, our study confirms the association between Lp(a) levels and the risk of CHD in T2DM patients.

## CONCLUSION

Our study highlights that Lp(a) is an independent risk factor for CHD in T2DM patients. Lp(a) levels have high clinical value in predicting the occurrence of CHD in T2DM patients. Lipid-lowering therapy with statins alone cannot reduce Lp(a) levels in these patients. Therefore, there is a pressing need to introduce effective medications and strategies to lower Lp(a) levels. These efforts are essential to mitigate the risk of cardiovascular events and improve clinical outcomes in T2DM patients.
